# Development and validation of an instrument in job evaluation factors of physicians in public hospitals in Beijing, China

**DOI:** 10.1371/journal.pone.0244584

**Published:** 2021-01-04

**Authors:** Dan Zhang, Meixia Liao, Pusheng Wang, Herng-Chia Chiu

**Affiliations:** 1 Institute for Hospital Management, Tsinghua University, Shenzhen, Guangdong, P. R. China; 2 Tsinghua Shenzhen International Graduate School, Tsinghua University, Shenzhen, Guangdong, P. R. China; 3 School of Social Sciences, Tsinghua University, Shenzhen, Guangdong, P. R. China; Imam Abdulrahman Bin Faisal University, SAUDI ARABIA

## Abstract

**Background:**

Job evaluation has been widely used to establish a foundation for internal equity and other human resource functions. The United Kingdom adopts the National Health Service (NHS) Job Evaluation scheme to determine the pay bands for most NHS staff and ensure equal pay for work of equal value. The challenges of recruiting and retaining physicians in Chinese public hospitals have heightened the need for a reliable job evaluation system to ensure the equality of physician compensation. The aim of this study was to construct job evaluation factors of physicians in Chinese public hospitals based on the NHS Job Evaluation scheme and to examine the reliability and validity of the established system.

**Methods:**

Questionnaire surveys and statistical analyses were used to determine the job evaluation factors for physicians. The preliminary screening of the evaluation factors was based on a literature review, focused interviews with physicians and the Delphi method. Based on the results of preliminary screening, a questionnaire with 25 factors was designed to survey physicians regarding the importance of each factor in physician job evaluation. After the pretest, final questionnaire data were collected from 900 physicians by adopting a stratified sampling from 6 tertiary public hospitals in Beijing. A principal component analysis was used for factor extraction and structural validity analysis. The reliability was determined using Cronbach’s alpha.

**Results:**

The results of the principal component analysis showed that the 25 physician job evaluation factors were grouped into the 5 dimensions of *Task Characteristics*, *Knowledge*, *Responsibility*, *Effort/Environment*, and *Skills*. The Cronbach’s alpha coefficients of the five dimensions ranged from 0.841 to 0.909, which indicated a high level of reliability. The result of the factor analysis indicated fair structural validity. The content validity was established by building onto the NHS Job Evaluation scheme and other well-established job evaluation systems.

**Conclusions:**

Our study indicates that the Chinese version of physician job evaluation is an instrument with fair reliability and validity, which fully reflects the characteristics of physicians in Chinese public hospitals. This system can provide an important basis for developing a physician compensation plan and ensuring internal equity in healthcare organizations.

## Introduction

A compensation plan is recognized as an essential method of attracting, retaining and motivating employees in an organization. It is important to develop an effective compensation programme by adopting an objective and systematic approach due to the complexity and subjectivity of compensation [1]. Job evaluation has been commonly used to establish a foundation for developing a fair wage structure and sustaining internal equity [2]. Specifically, job evaluation refers to a method for systematically determining the relative internal value of different jobs in an organization [2–4]. It can be used to design a fair payroll structure by comparing the relative similarities and differences in the content and the value of jobs. In addition to compensation management, job evaluation also serves as the basis for other human resource functions, such as recruitment, training, appraisal and career management [5].

There are several methods employed in job evaluation, including ranking, classification, factor comparison, job components and point systems [1]. The point-factor approach has been the most widespread method due to its comparatively high accuracy and reasonable cost [6,7]. The point-factor approach evaluates each position’s value through a set of compensable factors. These factors refer to the specific characteristics of the positions that contribute to the fulfilment of an organization’s strategy and objectives, and they are typically categorized as skills, responsibilities, effort and working conditions [4,8,9]. In this method, there is a scale defined by a number of levels for each of the factors, and each level is associated with a certain point value. The jobs are rated on all factors, and wage rates are assigned to the jobs based on the sum of all the factor scores [10].

Job evaluation, and in particular the point-factor method, has been widely used by a considerable number of companies and governments [11]. It is estimated that job evaluation has a major influence on the wages of half of American jobholders. For example, Mercer’s International Position Evaluation system with point-factor rating methodology has been used by multinational organizations to harmonize their compensation programmes [12]. The General Schedule developed by the U.S. Office of Personnel Management is the predominant pay scale for federal employees, including professional positions in healthcare organizations [13].

Job evaluation also has widespread usage in the field of healthcare. The National Health Service (NHS) Job Evaluation (JE) scheme has been the most significant change in human resource management in healthcare systems in the past decades [11]. The NHS JE scheme was developed by the Department of Health in the United Kingdom in 2003 and has been updated to the seventh edition [14]. It uses a common set of terms to describe jobs and produce national job profiles that are classified into the following eight professional groups: administrative services, allied health professionals, emergency services, health science services, nursing and midwifery, personal social services, support services and professional managers. The system provides a fair and clear method for determining the pay bands for all NHS staff, with the exception of doctors, dentists, apprentices and some senior managers, who have separate pay contracts [15]. The Review Body on Doctors’ and Dentists’ Remuneration (DDRB) brings an independent and expert view on rates of pay for medical and dental workforce whose salaries are based on years of experiences, merit awards, overtime and out-of-office hour payment [16]. Given that the internal relativities between medical and dental career paths is probably a controversial area, it is recommended that the relativities across different paths should be confirmed by a more detailed points factor job evaluation exercise, such as NHS JE system and Hay system [17]. NHS JE scheme focuses on the need to maintain equality and fairness and improve morale through better pay and overall quality. Previous research has assessed the quality of job evaluations and has mostly emphasized the issues of reliability and validity [1,10]. In addition, a number of studies have conducted a local evaluation of several healthcare jobs by implementing the NHS JE system to examine the appropriateness of the system in countries outside the United Kingdom [11,18].

Given the challenges of recruiting and retaining physicians and the fact that the public health sector is the main healthcare provider, physician compensation in public hospitals has been one of the core issues of China’s health system reforms [19]. Currently, Chinese public hospitals implement the Post-Performance Payment system to remunerate physicians, which consists of the basic salary, benefits and bonuses [20,21]. Following a unified standard, the basic salary is determined by physicians’ educational level, professional titles and years of practice. Benefits mainly consist of mandated benefits and supplemental welfare, such as medical insurance, endowment insurance, transportation subsidies and so on. The bonuses account for the majority of the total earnings and are wholly or partly bound to the revenues of the department [20]. Current system fails to consider other essential factors, such as the varying risks, responsibilities, skills and knowledge across different posts, which results in internal inequity and job dissatisfaction [22,23]. Moreover, physicians’ profit driven incentive is attributable to the bonuses linked to departmental revenues [20]. Job evaluation, especially the NHS JE system, is a promising method for addressing the internal inequity. The system that the basic pay makes up the majority of the total earnings for NHS staff also provides lessons for the physician compensation in Chinese public hospitals. However, all positions within an organization have their own characteristics. It is necessary to develop a tailor-made system to ensure that the system reflects local working practices. Furthermore, the selection of evaluation factors is a preliminary step in the design process of a point-factor job evaluation system. It is common to confirm the evaluation factors on the basis of the designer’s experience with and intentions for the management level, which causes a lack of theoretical support [24]. Therefore, to make the factors more reasonable and scientific, this study aims to develop the Chinese version of physician job evaluation (CN-PJE) system by building on the NHS JE system and adopting a questionnaire survey and to examine the reliability and validity of CN-PJE.

## Materials and methods

### Sample

In this study, we used a multistage sampling design to select the samples. In the first stage, we used cluster sampling to choose one tertiary public hospital within each district of Beijing, which resulted in six hospitals being included in our survey. Each of the participating hospitals had 800 to 1500 clinic beds and 1200 to 3500 employees. In the second stage, 5% of physicians were sampled within each of the participating hospitals using stratified random sampling and physicians were stratified based on the profession. A total of 900 physicians participated in the questionnaire survey.

### Questionnaire development

The selection of job evaluation factors for the questionnaire was based on a literature review, focused interviews with physicians and the Delphi method. First, through a literature analysis, we retained the 16 factors from the NHS JE system and consolidated another eight factors from four job evaluation models designed by governments or consulting firms [12,13,25,26]. Second, we identified an additional three factors through focused interviews with 20 physicians. Table 1 shows the lists of sources of job evaluation factors for the questionnaire.

**Table 1:** Lists of sources of job evaluation factors.

Number	Factors	M1	M2	M3	M4	M5		FI	D
1	Professional knowledge	√	√	√	√	√		√	√
2	Training	√				√		√	√
3	Experience	√		√		√		√	√
4	Communication and relationship skills	√	√	√	√	√		√	√
5	Planning and organizational skills	√		√		√		√	√
6	Analytical and judgemental skills	√		√		√		√	√
7	Physical skills	√		√		√		√	√
8	Responsibilities for patient/client care	√						√	√
9	Responsibilities for policy and service development and implementation	√	√	√		√		√	√
10	Responsibilities for financial and physical resources	√		√				√	√
11	Responsibilities for human resources	√			√	√		√	√
12	Responsibilities for information resources	√				√			√
13	Responsibilities for research and development	√						√	√
14	Freedom to act	√	√	√	√				√
15	Emotional effort	√				√		√	√
16	Mental effort	√						√	√
17	Physical effort	√	√					√	√
18	Working conditions	√	√	√	√	√		√	√
19	Innovation skills			√	√	√		√	√
20	Task relevance		√	√	√			√	√
21	Task complexity		√	√	√	√		√	√
22	Emergency response skills					√		√	√
23	Temporal characteristics					√		√	√
24	Knowledge updates					√		√	√
25	Awareness of quality and safety						√		√
26	Knowledge diversity							√	
27	Work urgency							√	

Note: National Health Service Job Evaluation scheme (M1) [14], Model by the U.S. Office of Personnel Management (M2) [13], Hay’s Job Evaluation Model (M3) [25], International Position Evaluation (IPE) (M4) [12], Model by Jinan Kangjia Hospital Management Consultants Ltd., (M5) [26], Focused Interviews (FI), Delphi method (D).

We screened and determined the factors using the two-round Delphi method with a panel of 15 experts. These experts included senior attending physicians and experts on hospital management and human resources. All of the experts were professors and had at least a Master’s degree. Most of the experts were 40 to 60 years old and had more than 20 years of work experience. We received 13 valid responses in the first round of the questionnaire and 12 valid responses in the second round. The result showed that the expert authority coefficient for all factors in the two rounds was higher than 0.8, suggesting that the result of the experts’ evaluation had high reliability. The Kendall coordination coefficient for all factors in the two rounds was statistically significant (*p*<0.05), which indicated that the result had high validity. After two rounds of Delphi, two factors, *Knowledge Diversity* and *Work Urgency*, were removed, and 25 factors were ultimately retained. The expert group used the Delphi process to derive a detailed definition of the following factors: *Analytical and Judgemental Skills*, *Task Complexity*, *Responsibilities for Policy and Service Development and Implementation*, *and Knowledge Updates*.

As shown in S1 and S2 Tables, the final version of the questionnaire contained 25 factors. The questionnaire asked participants to rate the importance of each factor on a 5-point Likert scale, ranging from “very important” (5 points) to “very unimportant” (1 point).

### Reliability and validity of the questionnaire

The questionnaire was pretested to assess its reliability and validity. A total of 50 physicians from a tertiary general hospital in Beijing participated in the preliminary survey. A total of 48 questionnaires were returned, with 45 valid responses. In the retest survey, 30 out of the 50 physicians accepted the retest administration within a two-week time span. The results showed that the overall Cronbach’s alpha of the questionnaire was 0.90, which implies satisfactory reliability. The test-retest reliability coefficient was 0.87, suggesting good retest reliability.

With the data from the preliminary survey, we applied Bartlett’s Test of Sphericity and the Kaiser-Meyer-Olkin (KMO) test to measure how suited the data are for factor analysis. The results showed that the Bartlett’s Test of Sphericity was significant at *p*< 0.05, and the KMO value was 0.879, which indicated that the data were appropriate for the purpose of factor analysis. We then used a principal component analysis to obtain the initial factor solutions. Five principal components with Eigenvalues greater than one were extracted, and they cumulatively explained 60.42% of the variance. Hence, the results indicated that the questionnaire had good structural validity.

### Data processing and statistical analysis

After receiving the completed questionnaires, a preprocessing step was applied to remove incomplete or invalid data. The exclusion criteria were as follows: 1) there were more than three factors unanswered; 2) all the factors were answered the same; and 3) the answers displayed an obvious pattern. In addition, after all the data had been entered, 5% of the data were drawn for rechecking to ensure the accuracy of data entry.

We performed a principal component analysis with a varimax rotation for feature extraction and dimensionality reduction. The factors with Eigenvalues greater than one were retained, and the factors with factor loadings less than 0.5 were removed from the analysis. The reliability of the job evaluation system was assessed by calculating Cronbach’s alpha and analysing the correlation between factors from the same and different dimensions. The validity was assessed via a structural validity test on the job evaluation factors. The data were processed with Microsoft Excel 2015 and analysed with SPSS 21.0.

### Ethics approval and consent to participate

This study has received ethics approval from Tsinghua Shenzhen International Graduate School Medical Ethics Committee. The questionnaire surveys were anonymous and voluntary, and written informed consent had been obtained from all study participants and experts.

## Results

### Characteristics of respondents

In this survey, a total of 900 questionnaires were distributed, and 759 were completed, which resulted in a response rate of 84.3%. A total of 693 questionnaires were regarded as valid, with a validity rate of 91.3%. Table 2 shows the characteristics of the study samples. Overall, 51.9% of the respondents were male, and 48.9% were aged between 30 and 39 years old. In terms of education, 51.7% had a Master’s degree. Attending physicians made up 47.8% of the sample. The majority of the respondents were general staff (70.7%) and had an annual salary of between 10,000 and 20,000 dollars (34.4%). In addition, most of the respondents worked in the surgery department (33.8%), followed by the internal medicine department (25.8%). There was no significant difference in the demographic characteristics between the exploratory samples and the confirmatory samples (*p*>0.05), which indicated that the responses had good consistency.

**Table 2:** Demographic characteristics of respondents (N = 693).

Variable	Items	Total (N = 693)		Exploratory Samples (N = 348)		Confirmatory Sample (N = 345)		*p*-value
		N	%	N	%	N	%	
Gender	Male	360	51.9	186	53.4	174	50.4	0.473
	Female	323	46.6	158	45.4	165	47.8	
	Missing	10	1.4	4	1.1	6	1.7	
Age (years)	< 30	101	14.6	49	14.1	52	15.1	0.597
	30–39	339	48.9	177	50.9	162	47.0	
	40–49	197	28.4	92	26.4	105	30.4	
	≥ 50	56	8.1	30	8.6	26	7.5	
Education level	Bachelor’s or below	134	19.3	68	19.5	66	19.1	0.212
	Master’s	358	51.7	180	51.8	178	51.6	
	PhD	201	29.0	100	28.7	101	29.3	
Title	Attending	331	47.8	167	48.0	164	47.5	0.927
	Junior attending	232	33.5	118	33.9	114	33.1	
	Senior attending	130	18.7	63	18.1	67	19.4	
Level of position	Head of department	71	10.2	32	9.2	39	11.3	0.332
	Group leader	132	19.1	70	20.1	62	18.0	
	General staff	490	70.7	246	70.7	244	70.7	
Working time (years)	≤5	128	18.5	60	17.2	68	19.7	0.454
	6–10	156	22.5	88	25.3	68	19.7	
	11–15	155	22.4	78	22.4	77	22.3	
	16–20	112	16.2	52	14.9	60	17.4	
	>20	142	20.5	70	20.1	72	20.9	
Annual income (dollars)	≤10,000	186	26.9	98	28.2	88	25.7	
	10,000–20,000	238	34.4	116	33.3	122	35.6	
	20,001–30,000	143	20.7	75	21.6	68	19.8	
	>30,000	124	17.9	59	17.0	65	19.0	
Departments	Internal medicine	179	25.8	89	25.6	90	26.1	0.683
	Surgery	234	33.8	117	33.6	117	33.9	
	Gynaecology and paediatrics	93	13.4	42	12.1	51	14.8	
	Emergency	85	12.3	43	12.4	42	12.2	
	Others	102	14.7	57	16.4	45	13.0	

### Descriptive statistics of the job evaluation factor questionnaire

As shown in Table 3, the average scores of the 25 factors ranged from 3.8 to 4.7, with a mean value of 4.2. The factor that received the highest score was *Professional Knowledge* (4.7±0.60), and the factors that received the lowest scores were *Responsibility for Financial and Physical Resources* (3.8±0.99) and *Responsibility for Information Resources* (3.8±0.87). The standard deviations were between 0.60 and 0.99, suggesting that all factors had a low degree of dispersion.

**Table 3:** Descriptive statistics of the job evaluation factor questionnaire (N = 693).

Factors	Minimum	Maximum	Mean	Standard deviation
Professional knowledge	4	5	4.7	0.60
Training	3	5	4.2	0.75
Experience	3	5	4.6	0.62
Communication and relationship skills	3	5	4.4	0.65
Planning and organizational skills	3	5	4.2	0.78
Analytical and judgemental skills	3	5	4.4	0.66
Physical skills	3	5	4.5	0.67
Responsibilities for patient/client care	3	5	4.2	0.82
Responsibilities for policy and service development and implementation	2	5	3.9	0.94
Responsibilities for financial and physical resources	1	5	3.8	0.99
Responsibilities for human resources	2	5	3.9	0.93
Responsibilities for information resources	1	5	3.8	0.87
Responsibilities for research and development	2	5	3.9	0.86
Freedom to act	3	5	4.2	0.82
Emotional effort	3	5	4.4	0.73
Mental effort	3	5	4.3	0.76
Physical effort	2	5	4.1	0.86
Working conditions	3	5	4.4	0.77
Innovation skills	2	5	4.1	0.84
Task relevance	2	5	4.1	0.77
Task complexity	2	5	4.1	0.81
Emergency response skills	3	5	4.4	0.71
Temporal characteristics	2	5	4.1	0.78
Knowledge updates	3	5	4.5	0.66
Awareness of quality and safety	3	5	4.4	0.78
**Mean**			4.2	0.77

### Factor analysis and dimensions

Bartlett’s Test of Sphericity was significant at *p*< 0.05, and the KMO value was 0.939, which supported the appropriateness of the principal component analysis technique. The principal components with Eigenvalues greater than one were retained. Five principal components were extracted from the principal component analysis and were defined as the five dimensions of the job evaluation system. The five components cumulatively explained 66.26% of the total variance, and the result was acceptable. A varimax rotation was used to transform the components into factors that were more clearly interpretable. Factors with factor loadings greater than 0.5 were extracted. All 25 factors had factor loadings above 0.5, and thus, all were retained in this study. Considering the content of the factors under each dimension, we named the five dimensions *Task Characteristics* (four factors), *Knowledge* (five factors), *Responsibility* (five factors), *Effort/Environment* (four factors) and *Skills* (seven factors). Table 4 demonstrates the factor loadings for each factor. The factor loadings of the factors in some dimensions were all above 0.7. For example, the five factors of the *Responsibility* dimension had factor loadings between 0.775 and 0.879, and the four factors of the *Effort/Environment* dimension had factor loadings between 0.734 and 0.843.

**Table 4:** Factor loadings by principal component analysis (N = 693).

Factor	Component				
	Task Characteristics	Knowledge	Responsibility	Effort/Environment	Skills
Task complexity	0.890	-	-	-	-
Task relevance	0.823	-	-	-	-
Temporal characteristics	0.804	-	-	-	-
Freedom to act	0.673	-	-	-	-
Professional knowledge	-	0.828	-	-	-
Knowledge updates	-	0.827	-	-	-
Physical skills	-	0.664	-	-	-
Experience	-	0.606	-	-	-
Awareness of quality and safety	-	0.563	-	-	-
Responsibilities for policy and service development and implementation	-	-	0.879	-	-
Responsibilities for financial resources	-	-	0.875	-	-
Responsibilities for human resources	-	-	0.857	-	-
Responsibilities for information resources	-	-	0.810	-	-
Responsibilities for research and development	-	-	0.775	-	-
Emotional effort	-	-	-	0.843	-
Mental effort	-	-	-	0.839	-
Physical effort	-	-	-	0.830	-
Working conditions	-	-	-	0.734	-
Planning and organizational skills	-	-	-	-	0.810
Communication and relationship skills	-	-	-	-	0.764
Analytical and judgemental skills	-	-	-	-	0.750
Emergency response skills	-	-	-	-	0.733
Innovation skills	-	-	-	-	0.715
Training	-	-	-	-	0.696
Responsibilities for patient/client care	-	-	-	-	0.687

### Reliability and validity analysis of the CN-PJE

The Cronbach’s alpha coefficients of the five dimensions ranged from 0.841 to 0.909, which indicated a high level of internal consistency. The Cronbach’s alphas were then calculated for each factor under the dimensions. As shown in Table 5, the removal of any factor would result in a lower Cronbach’s alpha. In terms of the corrected factor-total correlation, all correlation coefficients met the requirements, which suggested that the system’s factors and dimensions had good reliability. Moreover, the correlation coefficients between factors under the same dimension were all greater than 0.376, which indicated that the factors had good consistency, as shown in S3 Table.

**Table 5:** Reliability analysis of CN-PJE (N = 693).

Factor	Corrected factor-total correlation	Cronbach's Alpha if factor deleted	Cronbach's Alpha
Task complexity	0.870	0.882	0.907
Task relevance	0.823	0.888	
Temporal characteristics	0.804	0.895	
Freedom to act	0.673	0.906	
Professional knowledge	0.588	0.814	0.841
Knowledge updates	0.706	0.789	
Physical skills	0.681	0.810	
Experience	0.562	0.818	
Awareness of quality and safety	0.531	0.834	
Responsibilities for policy and service development and implementation	0.855	0.882	0.909
Responsibilities for financial and physical resources	0.847	0.889	
Responsibilities for human resources	0.801	0.890	
Responsibilities for information resources	0.768	0.892	
Responsibilities for research and development	0.703	0.905	
Emotional effort	0.795	0.860	0.885
Mental effort	0.746	0.846	
Physical effort	0.739	0.855	
Working conditions	0.699	0.874	
Planning and organizational skills	0.715	0.850	0.881
Communication and relationship skills	0.709	0.867	
Analytical and judgemental skills	0.717	0.858	
Emergency response skills	0.656	0.861	
Innovation skills	0.606	0.863	
Training	0.597	0.869	
Responsibilities for patient/client care	0.562	0.864	

Table 6 displays the inter-correlations of the five dimensions. The correlation coefficients between the dimensions were greater than 0.488, suggesting good consistency between the dimensions. *Knowledge* and *Skills* (r = 0.718) were the most correlated, while *Knowledge* and *Responsibility* (r = 0.488) were the least correlated.

**Table 6:** Inter-correlations of the five dimensions (N = 693).

Dimension (Mean/Standard Deviation)	Task Characteristics	Knowledge	Responsibility	Effort/Environment	Skills
Task Characteristics (4.1/0.66)	1	-	-	-	-
Knowledge (4.5/0.50)	0.570***	1	-	-	-
Responsibility (3.9/0.75)	0.608***	0.488***	1	-	-
Effort/Environment (4.2/0.65)	0.703***	0.524***	0.576***	1	-
Skills (4.3/0.57)	0.618***	0.718***	0.592***	0.496***	1

***denotes statistical significance at *p*<0.001.

The above results of the factor analysis show that the system has good structural validity. The content validity is well established by building on the NHS JE system and other well-established job evaluation systems with a two-round Delphi method. Therefore, our results show that the CN-PJE has both good reliability and validity.

## Discussion

In this study, we developed an instrument in job evaluation factors of physicians in public hospitals in Beijing, China. The CN-PJE was built on the NHS JE system with a two-round Delphi method and was further developed by a questionnaire survey to adjust for the differences in culture and healthcare delivery systems between these two countries. The CN-PJE consists of 25 factors scattered in five dimensions. It has high reliability, with Cronbach’s alphas all above 0.8. The results of the factor analysis show that the CN-PJE has sound structural validity. Therefore, the CN-PJE has both sound reliability and validity.

### The feature of CN-PJE

The survey results indicate that the CN-PJE can reflect the characteristics of physician posts. First, physicians are usually confronted with a number of challenges, including complicated clinical practices, varied clinical demands of patients, high risks, and heavy workloads [27]. These characteristics are reflected by the dimensions of *Task Characteristics* (four factors) and *Effort/Environment* (four factors). These factors should play an important role in determining a physician’s salary. Second, a physician is a knowledge-intensive position with high requirements for knowledge and skills [28,29]. These features are reflected by the dimensions of *Knowledge* (five factors) and *Skills* (seven factors). Third, physicians need to shoulder multiple responsibilities in hospitals [30], which can be captured by the *Responsibility* dimension (five factors). In addition to patient care, physicians also engage in management responsibilities, which involve policy development and implementation, financial and physical resources, human resources and information resources. Responsibility for research and development is an integral part of the work of physicians, especially for those in tertiary hospitals. Usually, research achievements can affect the salary of physicians in Chinese public hospitals. Furthermore, more responsibilities require more skills. Hospitals also put great value on physicians’ skills in problem solving, social interaction, administration and innovation. The survey suggests that physicians also attach importance to these skills and responsibilities.

The factors we developed in this study conform to the latest trends in job evaluation. To adapt to changes in the internal and external environments of hospitals, the job evaluation factors must reflect the organizational strategy and pay attention to the performance, contribution and development of employees [4]. Furthermore, job evaluation systems pay attention not only to fairness but also to flexibility [31]. These trends are embodied in this study. In conclusion, the CN-PJE can reasonably represent the relative values of physicians.

### The comparative analysis of two job evaluation systems

There are similarities and differences between the NHS JE and the CN-PJE, as shown in Table 7. Compared with the NHS JE system (five dimensions and 16 factors), the CN-PJE consists of similar dimensions but more factors (five dimensions and 25 factors). The factors of the *Effort/Environment* dimension are exactly the same, and the factors of *Responsibility* are almost the same as those of the NHS JE system. The results suggest that these dimensions and factors are reasonable.

**Table 7:** The dimensions and factors of CN-PJE and NHS JE.

Dimensions	CN-PJE	NHS JE
Task Characteristics	Freedom to act	Freedom to act
	Task complexity	*Newly added factor*
	Task relevance	*Newly added factor*
	Temporal characteristics	*Newly added factor*
Knowledge		Knowledge, training and experience
	Professional knowledge	*Split out from Knowledge*, *training and experience*
	Experience	*Split out from Knowledge*, *training and experience*
	Physical skills	*From Skills dimension*
	Knowledge update	*Newly added factor*
	Awareness of quality and safety	*Newly added factor*
Responsibility	Responsibility for policy and service development and implementation	Responsibility for policy and service development and implementation
	Responsibility for financial resources	Responsibility for financial resources
	Responsibility for human resources	Responsibility for human resources
	Responsibility for information resources	Responsibility for information resources
	Responsibility for research and development	Responsibility for research and development
		Responsibility for patient/client care
Effort/Environment	Emotional effort	Emotional effort
	Mental effort	Mental effort
	Physical effort	Physical effort
	Working conditions	Working conditions
Skills	Planning and organization skills	Planning and organization skills
	Communication and relationship skills	Communication and relationship skills
	Analytical and judgemental skills	Analytical and judgemental skills
	Emergency response skills	Physical skills
		*Newly added factor*
	Innovation skills	*Newly added factor*
	Training	*Split out from Knowledge*, *training and experience*
	Responsibility for patient/client care	*From Responsibility dimension*

In terms of differences, these two systems apply to different posts in healthcare organizations. The NHS JE scheme is used to determine the pay bands for all staff employed in the NHS, with the exception of doctors, dentists and some senior managers, who have separate pay contracts [14,15,32]. In contrast, the job evaluation factors in this study are specifically developed for physicians. Although the NHS JE system does not apply to physicians, it provides a rational framework for this study because most evaluation factors are common to all job positions. Moreover, significant effort has been made in this study to take into account the different aspects of physician posts so that the evaluation factors are adopted to the particular characteristics of physicians.

There are some differences in the factors of some dimensions. First, there are different numbers of factors under the *Knowledge* dimension. In the NHS JE system, there is only one factor under the *Knowledge* dimension to measure all the forms of knowledge required to fulfil the job responsibilities. This factor is the most heavily weighted factor (24%) and usually can make a difference between one pay band and the next [14]. In our study, we identified five factors to measure different forms of knowledge, specifically professional knowledge, experience, physical skills, awareness of quality and safety, and knowledge updates. Previous studies have suggested that having only one factor to measure all forms of knowledge might result in inaccurate and inconsistent outcomes [11,18]. Indeed, in addition to abundant professional knowledge, strong practical skills and rich experience [28], physicians need to have an awareness of quality and safety, given that patient safety is one of the most important components of health care delivery. In addition, physicians also need to constantly improve themselves through learning and updating new knowledge and techniques. Therefore, it is essential to define factors to measure each of these areas of knowledge.

Second, this study identified three new factors in the *Task Characteristics* dimension. *Task complexity* and *Task relevance* are mentioned by many other models, including the General Schedule of the U.S. Office of Personnel Management [13], Hay’s Job Evaluation model [25] and the International Position Evaluation model [12]. The amount of working time and duty periods vary from one post to another. Consequently, it is important to take into account the *temporal characteristics*. As suggested by our survey results, these new factors are highly accepted by physicians. In brief, these five factors can fully reflect the post characteristics of physicians.

Third, under the *Skills* dimension, *Emergency response skills* and *Innovation skills* are factors that are not included in the NHS JE system but are identified in our system. These factors can clearly reflect the skills required for physicians to address complicated medical services and the diversified clinical demands of patients. *Training* is from the *Knowledge* dimension, and *Responsibility for patient/client care* is from the *Skills* dimension of the NHS JE system. These two factors are the basis of other skills. Overall, the factors in this study are significantly correlated and can fully capture the characteristics of physicians.

### The application and implication of CN-PJE

The point-factor job evaluation is a systematic method for realizing internal equity within public organizations [11,33]. The current Post-Performance Payment system in Chinese public hospitals fails to consider internal relativities across different posts and results in internal inequity, job dissatisfaction and physicians’ profit driven incentive [20,22,23]. The CN-PJE system can be used in salary plans and to ensure internal equity for physicians. As the CN-PJE system is built on survey data from physicians, its design can better reflect what values physicians consider to be fair and will thus be better accepted by physicians. The CN-PJE has been adopted by two public hospitals in Beijing and have been used in their internal compensation programmes. Therefore, physician job evaluation can provide an important basis for the development of physician compensation plans. In addition, it can be combined with a variety of performance appraisal methods, such as the balanced scorecard and key performance indicators, to develop a reasonable salary system for physicians [34,35]. Furthermore, based on China’s explorations, the methods adopted by this study will be applicable to the localization of NHS JE and the establishment of job evaluation factors for other positions and in other countries.

### Limitations

Several limitations of this study should be mentioned. First, the samples for this study had a limited geographical range. As a result, this study can only represent the situation in Beijing and it can be viewed as a preliminary study. Further studies conducted in China’s different regions will reflect China’s whole situation and allow greater generalization. Second, the established CN-PJE has only been used in two hospitals in Beijing. The applicability of the system needs further verification through practice. Finally, this study was conducted in tertiary hospitals. Future studies can test the CN-PJE in other different levels of hospitals.

## Conclusions

This study developed job evaluation factors for physicians in public hospitals in Beijing, China. The CN-PJE system is an instrument with fair reliability and validity, consisting of five dimensions and 25 factors. These factors reflect the position characteristics of physicians in a comparatively complete and scientific manner. This system can provide an important basis for developing physician compensation plans and ensuring equal pay for work of equal value. The methods adopted by this study will be applicable to the establishment of job evaluation factors for other positions and other countries.

## Supporting information

S1 Table English version of the CN-PJE questionnaire. (DOC)

S2 Table Chinese version of the CN-PJE questionnaire. (DOC)

S3 Table Correlation analysis for physician job evaluation factors. (DOCX)

S1 Dataset Exploratory sample. (SAV)

S2 Dataset Confirmatory sample. (SAV)

## References

[1] Das B, Garcia-Diaz A. Factor selection guidelines for job evaluation: a computerized statistical procedure. Computers & Industrial Engineering. 2001; 40:259–272.

[2] Bosch T. Job evaluation revolution. Journal of Compensation and Benefits. 2015;31(3):33.

[3] Gupta S, Chakraborty M. Job evaluation in fuzzy environment. Fuzzy Sets Syst. 1998;100:71–76.

[4] Kutlu AC, Ekmekcioglu M, Kahraman C. A fuzzy multi-criteria approach to point-factor method for job evaluation. Journal of Intelligent& Fuzzy Systems. 2013;25:659–71.

[5] Doğan A, Önder E, Demir R. Assessment of Turkish HR professionals on determining the importance of factors in point factor as a method of job evaluation. Social Science Electronic Publishing. 2014;6(29):2222–2839.

[6] Plachy RJ, The case for effective point-factor job evaluation, viewpoint 1. Compens Benefits Rev. 1987;19(2):45–49.

[7] Candrilli AJ, Armagast RD. The case for effective point-factor job evaluation, viewpoint 2. Compens Benefits Rev. 1987;19(2):49–54.

[8] Milkovich GT, Newman JM, Gerhart B. Compensation. (11th ed). New York: McGraw Hill 2014.

[9] Berrocal FB, García MAA, Ramírez-Vielma R. Influence of the type of method on the results of job evaluation. UCJC Business and Society Review. 2018;15:114–146.

[10] Doverspike D, Carlisi AM, Barrett GV, et al Generalizability analysis of a point-method job evaluation instrument. J Appl Psychol. 1983;68(3):476–483.

[11] Kahya E, Oral N. Job evaluation for clinical nursing jobs by implementing the NHS JE system. J Nurs Manag. 2007;15:740–48. 10.1111/j.1365-2934.2006.00744.x 17897151

[12] Mercer enhances international position evaluation system to allow for easy evaluation of positions worldwide. 2008. https://www.prweb.com/releases/position/evaluation/prweb670354.htm. Accessed 9 July 2019.

[13] Job family position classification standard for professional work in the medical and healthcare group 0600. U.S. Office of Personnel Management; 2017.

[14] NHS Job Evaluation Handbook (Seventh Edition). London: British Department of Health Publication; 2019. https://www.nhsemployers.org/employershandbook/job-evaluation-handbook/job%20evaluation%20handbook.pdf. Accessed 9 July 2019.

[15] Buchan J, Evans D. Assessing the impact of a new health sector pay system upon NHS staff in England. Hum Resour Health. 2008;6(1):12 10.1186/1478-4491-6-12 18590569 PMC2475534

[16] Review Body on Doctors’ and Dentists’ Remuneration. Review Body on Doctors’ and Dentists’ Remuneration Forty-Eighth Report 2020. 2020. https://www.gov.uk/government/publications/review-body-on-doctors-and-dentists-remuneration-48th-report-2020. Accessed 20 October 2020.

[17] Institute for Employment Studies. Review of DDRB pay comparability methodology: final report. 2017. https://assets.publishing.service.gov.uk/government/uploads/system/uploads/attachment_data/file/637298/Review_of_DDRB_Pay_Comparability_Methodology_Final_Report_2017.pdf. Accessed 20 October 2020.

[18] Kahya E. A revision of a job evaluation system. J Adv Nurs. 2006;56(3):314–324. 10.1111/j.1365-2648.2006.04024.x 17042810

[19] Zhang C, Liu Y. The salary of physicians in Chinese public tertiary hospitals: a national cross-sectional and follow-up study. BMC Health Serv Res. 2018;18:661 10.1186/s12913-018-3461-7 30143042 PMC6109324

[20] Ran LM, Luo KJ, Wu YC, et al An analysis of China's physician salary payment system. J Huazhong Univ Sci Technolog Med Sci. 2013;33:309–14. 10.1007/s11596-013-1116-9 23592149

[21] Feng HJ, Hao ZM. The reform on physician payment of Chinese public hospitals since the founding of The People’s Republic of China. China Health Human Resources. 2019;7:58–61. (in Chinese).

[22] Wang YZ, Hou JL. Issues and reform suggestions for existing problems in payment policy of public hospitals in China. Chinese Health Economics. 2015;34(1):5–8. (in Chinese).

[23] Zhang C, Liu Y. The salary of physicians in Chinese public tertiary hospitals: a national cross-sectional and follow-up study. *BMC Health Serv Res*. 2018;18(1):661 10.1186/s12913-018-3461-7 30143042 PMC6109324

[24] Sun X, Luo N. Study on the effectiveness of point-factor job evaluation system in operation position. Communications in Information Science and Management Engineering. 2013;3(3):154–160.

[25] Johnson P, McMullen T, Royal M. Job evaluation: relevant, robust and reimagined. Workspan. 2015;26.

[26] Jinan Kangjia Hospital Management Consulting Co., Ltd. Success application of job evaluation in consultation for hospital human resource management. 2010. http://www.jnkhmc.com/newsShow.asp?newsTypeID=33&ID=674. Accessed 9 July 2019.

[27] Chen HL, Yang MC, Wen HC. Physician workload relative value scale and procedure duration for 86 gastroenterologic surgery treatments in Taiwan. Journal of Health Science. 2005;7(2):175–85.

[28] Hsiao WC, Braun P, Becker ER, Thomas SR. The resource-based relative value scale-toward the development of an alternative physician payment system. JAMA. 1987;258(6)799–802. 3613008

[29] Laugesen MJ. The resource-based relative value scale and physician reimbursement policy. Chest. 2014;146(5):1413–19. 10.1378/chest.13-2367 25367477

[30] Nistor DL. The analysis of the representativeness of results obtained after applying the method of job evaluation through tasks. Theoretical and Applied Economics. 2012;3(568):121–36.

[31] Pandey J. A study on job evaluation-point factor analysis in SME'S. Asian Journal of Research in Business Economics and Management. 2012;2(5):179–239.

[32] Buchan J, Ball J. Evaluating the impact of a new pay system on nurses in the UK. J Clin Nurs. 2011;20(1–2):50–9. 10.1111/j.1365-2702.2010.03310.x 21040031

[33] Wall D, Goodyear H, Singh B, Whitehouse A, Hughes E, Howes J. A new tool to evaluate postgraduate training posts: the Job Evaluation Survey Tool (JEST). BMC Med Educ. 2014;14:210 10.1186/1472-6920-14-210 25277827 PMC4200137

[34] Rollie W, Cox TL. Public sector compensation panacea or Pandora’s box? Public management. 2011;93(10):13–15.

[35] Wang Y, Zhang D, Xu S, Zhang T. Establishment of doctor-oriented job evaluation factor system for general hospitals and survey analyses on its reliability and validity. Journal of Jilin University (Medicine Edition). 2014;40(6):1308–1313. (in Chinese).

