# Supplementary material for: Development and validation of an instrument in job evaluation factors of physicians in public hospitals in Beijing, China
Source: PLoS One. 2021 Jan 4;16(1):e0244584. doi: 10.1371/journal.pone.0244584 (PMC7781376; doi:10.1371/journal.pone.0244584)
Supplement: S1 Table — (DOC) [file pone.0244584.s001.doc]

**Questionnaire on physician job evaluation factors in public hospitals**

Job evaluation serves as the basis of job management and performance based wage system. Determining job evaluation factors in a scientific and reasonable manner is the prerequisite for job evaluation. The purpose of this survey is to understand which factors can best reflect the value of jobs from the perspective of physicians in public hospitals, and to build a physician job evaluation system for public hospitals in China.

The following lists 25 job evaluation factors related to physicians. Based on your profession and work experience, please rate the importance of each factor and tick the corresponding item using "√" . If there are factors that can reflect the value of jobs but are not list below, please fill in the black space and rate their importance.

All information in this questionnaire is for scientific research only, and the survey data will be strictly confidential. The research results do not involve any personal information. The credibility of the research results depends on your serious and objective answers to the questions. When you fill out this questionnaire, please read the questions carefully and truly express your views. The information you provide will be of great help to our research. Contact tel: 18664555188; Email: zd52015@163.com.

Thank you for your cooperation and contribution to this research.

Based on your profession and work experience, please rate the importance of each factor and tick the corresponding item using "√" .

| **No.** | **Job evaluation factor and its definition** | **Very unimportant** | **Unimportant** | **Neutral** | **Important** | **Very important** |  |
| --- | --- | --- | --- | --- | --- | --- | --- |
| **1** | **Professional knowledge**  This factor measures all the forms of knowledge required to fulfil the job responsibilities. | **1**  □ | **2**  □ | **3**  □ | **4**  □ | **5**  □ |  |
| **2** | **Knowledge updates**  This factor measures the ability to actively expand learning channels, master scientific learning methods, constantly update and improve the knowledge structure, and improve work ability. | **1**  □ | **2**  □ | **3**  □ | **4**  □ | **5**  □ |  |
| **3** | **Experience**  This factor measures the relevant practice and work experience required to fulfil the job responsibilities. | **1**  □ | **2**  □ | **3**  □ | **4**  □ | **5**  □ |  |
| **4** | **Training**  This factor measures the systematic and standardized training required to fulfil the job responsibilities. | **1**  □ | **2**  □ | **3**  □ | **4**  □ | **5**  □ |  |
| **5** | **Communication and relationship skills**  This factor measures the ability to accurately understand the thoughts and intentions of patients or others, and to choose appropriate communication methods to communicate effectively. The ability to reasonably use interpersonal skills in interpersonal communication, to promote mutual understanding and trust, and to build good interpersonal relationship. | **1**  □ | **2**  □ | **3**  □ | **4**  □ | **5**  □ |  |
| **5** | **Planning and organizational skills**  This factor measures the ability to formulate a scientific work plan, effectively arrange the work process, and rationally allocate various resources to ensure the efficient and orderly completion of various tasks. | **1**  □ | **2**  □ | **3**  □ | **4**  □ | **5**  □ |  |
| **7** | **Innovation skills**  This factor measures the ability to keep pace with the times, be good at discovering new things, propose new ideas, solve new problems, summarize new experiences, and combine practical work creatively with an innovative spirit. | **1**  □ | **2**  □ | **3**  □ | **4**  □ | **5**  □ |  |
| **8** | **Analytical and judgemental skills**  This factor measures the ability to observe, analyze, distinguish and research systematically and objectively, and to make reasonable judgments and reasoning. | **1**  □ | **2**  □ | **3**  □ | **4**  □ | **5**  □ |  |
| **9** | **Emergency response skills**  This factor measures the ability to grasp and prevent potential problems in a timely manner, formulate feasible plans, keenly grasp the potential impact of events, quickly respond, take effective response measures, and properly solve the problem. | **1**  □ | **2**  □ | **3**  □ | **4**  □ | **5**  □ |  |
| **10** | **Physical skills**  This factor measures the physical skills required in the process of diagnosis, treatment, nursing, surgery or other operation. | **1**  □ | **2**  □ | **3**  □ | **4**  □ | **5**  □ |  |
| **11** | **Responsibilities for patient/client care**  This factor measures the responsibilities for patient’s diagnosis, treatment, nursing and other services, which are mainly assessed according to the degree of participation and responsibility sharing. | **1**  □ | **2**  □ | **3**  □ | **4**  □ | **5**  □ |  |
| **12** | **Awareness of quality and safety**  This factor measures the duties to ensure medical quality and safety, prevent and control medical risks, reduce medical disputes, and safeguard the legitimate rights and interests of patients. | **1**  □ | **2**  □ | **3**  □ | **4**  □ | **5**  □ |  |
| **13** | **Responsibilities for research and development**  This factor measures the responsibilities to learn and use advanced technology at home and abroad, and to organize, guide or participate in clinical or non-clinical scientific research. | **1**  □ | **2**  □ | **3**  □ | **4**  □ | **5**  □ |  |
| **14** | **Responsibilities for policy and service development and implementation**  This factor measures the responsibilities to participate in the hospital's managerial decision-making and its implementation and to take the responsibility for corresponding results. The assessment is mainly based on the level of participation in decision-making and influence of the decision-making results on the hospital. | **1**  □ | **2**  □ | **3**  □ | **4**  □ | **5**  □ |  |
| **15** | **Responsibilities for human resources**  This factor measures the responsibilities to put forward specific opinions or carry out responsibilities in terms of the hospital personnel’s planning, selection, employment, assessment, and incentives. | **1**  □ | **2**  □ | **3**  □ | **4**  □ | **5**  □ |  |
| **16** | **Responsibilities for financial and physical resources**  This factor measures the responsibilities of hospital financial management and asset control. Financial management includes the issuance of cash or financial payments, budget formulation and expenditure, and financial review. Asset control includes the management responsibilities of tangible and intangible assets. | **1**  □ | **2**  □ | **3**  □ | **4**  □ | **5**  □ |  |
| **17** | **Responsibilities for information resources**  This factor measures responsibilities of the information recording, collection, storage, transmission, sharing, confidentiality, processing, and maintenance of hospital information systems. | **1**  □ | **2**  □ | **3**  □ | **4**  □ | **5**  □ |  |
| **18** | **Physical effort**  This factor measures the level of physical effort required for the job. It takes account of working posture, and frequency and duration of work. | **1**  □ | **2**  □ | **3**  □ | **4**  □ | **5**  □ |  |
| **19** | **Mental effort**  This factor measures the level of mental effort required for the job. It takes account of the nature, frequency, level and duration of mental effort and anti-interference ability. | **1**  □ | **2**  □ | **3**  □ | **4**  □ | **5**  □ |  |
| **20** | **Emotional effort**  This factor measures the level of the psychological pain and emotional fluctuations suffered from the job, such as facing the death or illness of the patient. It takes account of the nature, frequency, level and duration of emotional effort required for the job. | **1**  □ | **2**  □ | **3**  □ | **4**  □ | **5**  □ |  |
| **21** | **Working conditions**  This factor measures the level of danger (such as infection, occupational injury, etc.) and discomfort (such as noise, radiation, dust, etc.) arising from the working conditions. | **1**  □ | **2**  □ | **3**  □ | **4**  □ | **5**  □ |  |
| **22** | **Freedom to act**  This factor measures the level of freedom of the job. It takes account of the degree of job standardization, and the type and frequency of supervision and guidance required for the job. | **1**  □ | **2**  □ | **3**  □ | **4**  □ | **5**  □ |  |
| **23** | **Task relevance**  This factor measures the level of task relevance with others. It takes account of the degree and scope of the influence on others due to the delay or mistakes while undertaking the tasks. | **1**  □ | **2**  □ | **3**  □ | **4**  □ | **5**  □ |  |
| **24** | **Task complexity**  This factor measures the complexity of the job and the difficulty of performing the responsibilities. | **1**  □ | **2**  □ | **3**  □ | **4**  □ | **5**  □ |  |
| **25** | **Temporal characteristics**  This factor measures the specific starting and ending time of the job, the regularity of working hours, and the frequency of business trips, overtime or on duty. | **1**  □ | **2**  □ | **3**  □ | **4**  □ | **5**  □ |  |
| If there are factors that can reflect the value of jobs but are not list below, please fill in the black space and rate their importance. | | | | | | | |
| **A** |  | **1**  □ | **2**  □ | **3**  □ | **4**  □ | **5**  □ |  |
| **B** |  | **1**  □ | **2**  □ | **3**  □ | **4**  □ | **5**  □ |  |
| **C** |  | **1**  □ | **2**  □ | **3**  □ | **4**  □ | **5**  □ |  |
| **D** |  | **1**  □ | **2**  □ | **3**  □ | **4**  □ | **5**  □ |  |
| **E** |  | **1**  □ | **2**  □ | **3**  □ | **4**  □ | **5**  □ |  |

**Personal information**

Please tick the corresponding item using “√”.

| Gender： □ Male（1） □ Female（2）  Age： □ Below 30 years old（1） □ 30～39 years old（2） □ 40～50 years old（3） □ above 50 years old（4）  Education level： □ Below Bachelor’s degree（1） □ Bachelor’s degree（2） □ Master’s degree（3） □ Phd（4）  Title： □ Attending（1） □ Junior attending（2） □ Senior attending（3）  Working time：□ 1～5 years （1） □ 6～10 years（2） □ 11～15 years（3） □ 16～20 years（4）  □ 21～25 years（5） □ 26～30 years（6） □ above 30 years（7）  Level of position：□ Head of hospital（1） □ Head of department（2） □ Head of unit（3）  □ Group leader（4） □ General staff（5）  Monthly income：□ Below 4000 Yuan（1） □ 4001～6000 Yuan（2）  □ 6001～8000 Yuan（3） □ 8001～10000 Yuan（4） □ Above 10000 Yuan（5）  Hospital： □ A hospital（1） □ B hospital （2）□ C hospital（3） □ D hospital（4） □ E hospital（5） □ F hospital（6）  Department：□ Radiology（1） □ Pharmacy（2） □ Endocrinology（3） □ Ophthalmology（4） □ Laboratory（5）  □ Dermatology（6） □ General Surgery（7） □ Gynecology and Obstetrics（8） □ Emergency（9） □ Cardiology（10）  □ Pediatrics（11） □ Orthopedics（12） □ Cath lab（13） □ Psychiatry (14) □ Stomatology (15) □ Rehabilitation (16)  □ Traditional Chinese Medicine (17) □ General Internal Medicine (18) □ Otorhinolaryngology (19) □ Anesthesiology (20)  □ Pathology (21) □ Nutrition (22) □ ICU (23) |
| --- |

Thank you for your cooperation and support.
