# Supplementary material for: Development and validation of an instrument in job evaluation factors of physicians in public hospitals in Beijing, China
Source: PLoS One. 2021 Jan 4;16(1):e0244584. doi: 10.1371/journal.pone.0244584 (PMC7781376; doi:10.1371/journal.pone.0244584)
Supplement: S2 Table — (DOC) [file pone.0244584.s002.doc]

**公立医院医师岗位评价要素调查问卷**

岗位评价是推行岗位管理制度和岗位绩效工资制度的基础，而确定科学合理的岗位评价要素是岗位评价工作的前提。本次调查的目的是了解在公立医院职工的心目中，与工作岗位相关的哪些要素最能体现岗位的价值，据此构建中国公立医院岗位评价要素模型。

以下列出了与医院岗位相关的25项岗位评价要素，请您结合自己的工作岗位和实际工作经验，评价各项要素对于岗位的重要程度，并在相应的重要程度上打钩（“√”）。如果下表中未列出您所认为的能反映岗位价值的评价要素，请您在每部分后面的空白处填写，并填写该要素的重要程度。

本问卷中所有资料只作科学研究，调查资料将会严格保密，研究结果只展现综合数据，不涉及任何个人信息。研究结果的可信度取决于您对问题认真和客观的回答，请您填写此问卷时，细心阅读各项问题，真实地表达您的看法和意见。您所提供的信息对我们的研究会有很大的帮助。咨询电话： 18664555188；邮箱：zd52015@163.com。

感谢您的配合和对公立医院岗位评价要素研究的贡献!

**请您结自己的工作岗位和实际工作经验，**评价各项要素对于岗位的重要程度,在相应的重要程度上打钩，并只能选择其中壹项。

| **序号** | **岗位评价要素及其释义** | **非常不重要** | **不重要** | **壹般** | **重要** | **非常重要** |
| --- | --- | --- | --- | --- | --- | --- |
| **1** | **专业技术知识**  指任职者胜任岗位工作所需要掌握的系统化的专业理论知识与技术。 | **1**  □ | **2**  □ | **3**  □ | **4**  □ | **5**  □ |
| **2** | **知识更新**  指任职者胜任岗位工作需要积极拓展学习途径，掌握科学的学习方法，不断更新和完善知识结构、提升工作能力的要求。 | **1**  □ | **2**  □ | **3**  □ | **4**  □ | **5**  □ |
| **3** | **经验**  指任职者胜任岗位工作所需要的相关社会实践与工作经历。 | **1**  □ | **2**  □ | **3**  □ | **4**  □ | **5**  □ |
| **4** | **培训**  指任职者胜任岗位工作所需要接受的系统规范化培训。 | **1**  □ | **2**  □ | **3**  □ | **4**  □ | **5**  □ |
| **5** | **沟通与人际关系能力**  指任职者能够准确理解患者或他人的想法和意图，并选择适当的沟通方式，进行有效沟通的能力，以及任职者在人际交往中，合理运用人际关系处理技巧，促进相互理解与信任，建立良好人际关系的能力。 | **1**  □ | **2**  □ | **3**  □ | **4**  □ | **5**  □ |
| **5** | **计划与组织能力**  指任职者能够在工作前制定科学的工作计划，有效安排工作进程，并在工作过程中合理调配各种资源，确保各项工作高效有序完成的能力。 | **1**  □ | **2**  □ | **3**  □ | **4**  □ | **5**  □ |
| **7** | **创新能力**  指任职者能够解放思想，与时俱进，以创新的精神和务实的态度，善于发现新事物，提出新思路，解决新问题，总结新经验，并结合实际创造性地开展工作的能力。 | **1**  □ | **2**  □ | **3**  □ | **4**  □ | **5**  □ |
| **8** | **分析能力**  指任职者能够全面、系统、客观地进行观察、剖析、分辨和研究，做出合理判断推理的能力。 | **1**  □ | **2**  □ | **3**  □ | **4**  □ | **5**  □ |
| **9** | **应对突发事件能力**  指任职者能够及时掌握并防范潜在性问题，制定可行预案，并在面对突发事件时，能够敏锐把握事件潜在影响，迅速做出正确反应，采取有效的应对措施，予以妥善解决的能力。 | **1**  □ | **2**  □ | **3**  □ | **4**  □ | **5**  □ |
| **10** | **操作能力**  指在诊疗、护理、手术或仪器操作等过程中对任职者操作技能水平的要求。 | **1**  □ | **2**  □ | **3**  □ | **4**  □ | **5**  □ |
| **11** | **患者服务职责**  指任职者在岗位工作中所承担的对患者进行诊疗、护理以及其他服务的职责，主要根据参与的程度以及职责分担程度来评定。 | **1**  □ | **2**  □ | **3**  □ | **4**  □ | **5**  □ |
| **12** | **质量安全职责**  指任职者在岗位工作中所承担的保障医疗质量与安全，防范与控制医疗风险，减少医疗纠纷，维护患者合法权益的职责。 | **1**  □ | **2**  □ | **3**  □ | **4**  □ | **5**  □ |
| **13** | **科学研究职责**  指任职者能够学习与运用国内外先进科学技术，组织、指导或参与临床或非临床科研工作的职责。 | **1**  □ | **2**  □ | **3**  □ | **4**  □ | **5**  □ |
| **14** | **管理决策与实施职责**  指任职者在工作中参与医院的管理决策及其实施，以及承担相应工作结果的职责。主要根据任职者参与决策的层次和决策结果对医院影响的程度来评定。 | **1**  □ | **2**  □ | **3**  □ | **4**  □ | **5**  □ |
| **15** | **人员管理职责**  指任职者在工作中所承担的医院人员规划、选拔、聘用、考核、激励等方面提出具体意见或进行实施的职责。 | **1**  □ | **2**  □ | **3**  □ | **4**  □ | **5**  □ |
| **16** | **财务与资产控制职责**  指任职者在工作中所承担的医院财务管理与资产控制的职责。其中财务管理包括现金或财务支付的签授、预算的制定与支出、财务审查等。资产控制包括任职者所要承担的有形资产与无形资产的管理责任。 | **1**  □ | **2**  □ | **3**  □ | **4**  □ | **5**  □ |
| **17** | **信息资源管理职责**  指任职者在工作中所承担的信息记录、收集、存储、传递、共享、保密、处理，以及医院信息系统的维护等职责。 | **1**  □ | **2**  □ | **3**  □ | **4**  □ | **5**  □ |
| **18** | **体力强度**  指任职者在工作中体力支出的水平。主要根据工作时的工作姿势、用力大小和持续时间来评定。 | **1**  □ | **2**  □ | **3**  □ | **4**  □ | **5**  □ |
| **19** | **脑力强度**  指任职者在工作中所需要的注意力集中程度。主要根据集中精力的时间、频率、水平和抗干扰能力等方面来评定。 | **1**  □ | **2**  □ | **3**  □ | **4**  □ | **5**  □ |
| **20** | **心理与精神强度**  指任职者在工作中所承受的心理痛苦以及情绪波动程度。如面对患者死亡或病痛等。主要根据其强度的大小、持续的时间、频率等方面来评定。 | **1**  □ | **2**  □ | **3**  □ | **4**  □ | **5**  □ |
| **21** | **工作环境**  指工作环境中存在的危险性（感染、职业损伤等）和不适性（噪音、辐射、粉尘等）的程度。 | **1**  □ | **2**  □ | **3**  □ | **4**  □ | **5**  □ |
| **22** | **工作自主性**  指任职者在工作过程中自主权的大小。主要根据岗位工作的规范程度，以及任职者所受到的监督和指导类型及其频率来评定。 | **1**  □ | **2**  □ | **3**  □ | **4**  □ | **5**  □ |
| **23** | **工作关联性**  指岗位工作与他人工作的关联程度，主要根据任职者因承办事务延误或失误对他人工作的影响程度和范围来评定。 | **1**  □ | **2**  □ | **3**  □ | **4**  □ | **5**  □ |
| **24** | **工作复杂性**  指任职者所承担工作任务的复杂程度，以及履行职责的难易程度。 | **1**  □ | **2**  □ | **3**  □ | **4**  □ | **5**  □ |
| **25** | **工作时间特性**  指岗位工作特定的起止时间，工作时间的规律性，以及需要出差、加班或值班的频率。 | **1**  □ | **2**  □ | **3**  □ | **4**  □ | **5**  □ |
| **如上表中未能列出您所认为的岗位评价要素，请您在以下空白处填写，并填写出您所认为该要素的重要程度：** | | | | | | |
| **A** |  | **1**  □ | **2**  □ | **3**  □ | **4**  □ | **5**  □ |
| **B** |  | **1**  □ | **2**  □ | **3**  □ | **4**  □ | **5**  □ |
| **C** |  | **1**  □ | **2**  □ | **3**  □ | **4**  □ | **5**  □ |
| **D** |  | **1**  □ | **2**  □ | **3**  □ | **4**  □ | **5**  □ |
| **E** |  | **1**  □ | **2**  □ | **3**  □ | **4**  □ | **5**  □ |
| **个人基本资料**  请您在相应的选项前打钩（“√”） | | | | | | |
| **性别：** □男（1） □女（2）  **年龄：** □30岁以下（1） □30～39岁（2） □40～50岁（3） □50岁以上（4）  **学历：** □大专及以下（1） □大学本科（2） □硕士（3） □博士（4）  **职称：** □初级（1） □中级（2） □正高（3）  **工作年限：**□1～5年 （1） □6～10年（2） □11～15年（3） □16～20年（4）  □21～25年（5） □26～30年（6） □30年以上（7）  **岗位级别：**□院级领导（1） □科主任（含副职）（2） □职能处室负责人（含副职）（3）  □科长等基层领导（4） □普通医师（5）  **每月收入：**□4000元以下（1） □4001～6000元（2）  □6001～8000元（3） □8001～10000元（4） □10000元以上（5）  **所属医院：** □A医院（1） □B医院 （2）□C医院（3） □D医院（4） □E医院（5） □F医院（6）  **所在科室：**□放射科（1） □药剂科（2） □内分泌科（3） □眼科（4） □检验科（5）  □皮肤科（6） □普外科（7） □妇产科（8） □急诊科（9） □心内科（10）  □儿科（11） □骨科（12） □导管室（13） □精神科（14）□口腔科（15）  □康复科（16） □中医科（17） □普内科（18） □耳鼻喉科（19）□麻醉科（20）  □病理科（21） □营养科（22） □各类ICU（23） | | | | | | |

**衷心感谢您的配合与支持！**
