# Supplementary material for: Development and validation of an instrument in job evaluation factors of physicians in public hospitals in Beijing, China
Source: PLoS One. 2021 Jan 4;16(1):e0244584. doi: 10.1371/journal.pone.0244584 (PMC7781376; doi:10.1371/journal.pone.0244584)
Supplement: S3 Table — (DOCX) [file pone.0244584.s003.docx]

**S3 Table** Correlation analysis for physician job evaluation factors (N=693)

| Items | TC | TR | TE | FA | PK | KU | PS | E | QS | DI | FA | HR | IM | RD | EE | ME | PE | WE | PO | CR | AS | ER | IS | T | PC |
| --- | --- | --- | --- | --- | --- | --- | --- | --- | --- | --- | --- | --- | --- | --- | --- | --- | --- | --- | --- | --- | --- | --- | --- | --- | --- |
| Task complexity | 1 |  |  |  |  |  |  |  |  |  |  |  |  |  |  |  |  |  |  |  |  |  |  |  |  |
| Task relevance | 0.662 | 1 |  |  |  |  |  |  |  |  |  |  |  |  |  |  |  |  |  |  |  |  |  |  |  |
| Temporal characteristics | 0.684 | 0.551 | 1 |  |  |  |  |  |  |  |  |  |  |  |  |  |  |  |  |  |  |  |  |  |  |
| Freedom to act | 0.518 | 0.594 | 0.500 | 1 |  |  |  |  |  |  |  |  |  |  |  |  |  |  |  |  |  |  |  |  |  |
| Professional knowledge |  |  |  |  | 1 |  |  |  |  |  |  |  |  |  |  |  |  |  |  |  |  |  |  |  |  |
| Knowledge updates |  |  |  |  | 0.524 | 1 |  |  |  |  |  |  |  |  |  |  |  |  |  |  |  |  |  |  |  |
| Physical skills |  |  |  |  | 0.458 | 0.495 | 1 |  |  |  |  |  |  |  |  |  |  |  |  |  |  |  |  |  |  |
| Experience |  |  |  |  | 0.437 | 0.507 | 0.526 | 1 |  |  |  |  |  |  |  |  |  |  |  |  |  |  |  |  |  |
| Awareness of quality and safety |  |  |  |  | 0.376 | 0.444 | 0.407 | 0.387 | 1 |  |  |  |  |  |  |  |  |  |  |  |  |  |  |  |  |
| Responsibilities for policy and service development and implementation |  |  |  |  |  |  |  |  |  | 1 |  |  |  |  |  |  |  |  |  |  |  |  |  |  |  |
| Respponsibilities for financial and physical resources |  |  |  |  |  |  |  |  |  | 0.703 | 1 |  |  |  |  |  |  |  |  |  |  |  |  |  |  |
| Responsibilities for human resources |  |  |  |  |  |  |  |  |  | 0.782 | 0.716 | 1 |  |  |  |  |  |  |  |  |  |  |  |  |  |
| Responsibilities for information resources |  |  |  |  |  |  |  |  |  | 0.649 | 0.736 | 0.594 | 1 |  |  |  |  |  |  |  |  |  |  |  |  |
| Responsibilities for research and development |  |  |  |  |  |  |  |  |  | 0.660 | 0.550 | 0.582 | 0.583 | 1 |  |  |  |  |  |  |  |  |  |  |  |
| Emotional effort |  |  |  |  |  |  |  |  |  |  |  |  |  |  | 1 |  |  |  |  |  |  |  |  |  |  |
| Mental effort |  |  |  |  |  |  |  |  |  |  |  |  |  |  | 0.703 | 1 |  |  |  |  |  |  |  |  |  |
| Physical effort |  |  |  |  |  |  |  |  |  |  |  |  |  |  | 0.651 | 0.732 | 1 |  |  |  |  |  |  |  |  |
| Working conditions |  |  |  |  |  |  |  |  |  |  |  |  |  |  | 0.507 | 0.539 | 0.513 | 1 |  |  |  |  |  |  |  |
| Planning and organizational skills |  |  |  |  |  |  |  |  |  |  |  |  |  |  |  |  |  |  | 1 |  |  |  |  |  |  |
| Communication and relationship skills |  |  |  |  |  |  |  |  |  |  |  |  |  |  |  |  |  |  | 0.571 | 1 |  |  |  |  |  |
| Analytical and judgmental skills |  |  |  |  |  |  |  |  |  |  |  |  |  |  |  |  |  |  | 0.581 | 0.462 | 1 |  |  |  |  |
| Emergency response skills |  |  |  |  |  |  |  |  |  |  |  |  |  |  |  |  |  |  | 0.556 | 0.502 | 0.609 | 1 |  |  |  |
| Innovation skills |  |  |  |  |  |  |  |  |  |  |  |  |  |  |  |  |  |  | 0.642 | 0.412 | 0.584 | 0.480 | 1 |  |  |
| Training |  |  |  |  |  |  |  |  |  |  |  |  |  |  |  |  |  |  | 0.545 | 0.527 | 0.445 | 0.428 | 0.431 | 1 |  |
| Responsibilities for patient/client care |  |  |  |  |  |  |  |  |  |  |  |  |  |  |  |  |  |  | 0.513 | 0.466 | 0.541 | 0.552 | 0.503 | 0.461 | 1 |

Note: All correlations are significant at *p*<0.001.
